# Supplementary material for: Global profiling of regulatory elements in the histone benzoylation pathway
Source: Nat Commun. 2022 Mar 16;13:1369. doi: 10.1038/s41467-022-29057-2 (PMC8927147; doi:10.1038/s41467-022-29057-2)
Supplement: Supplementary file 1 — Supplementary Information [file 41467_2022_29057_MOESM1_ESM.pdf]

**Supplementary information**

# Global profiling of regulatory elements in the histone benzoylation pathway

Duo Wang[1,2,#], Fuxiang Yan[1,2,#], Ping Wu[3,#], Kexue Ge[1,2], Muchun Li[1,2,4], Tingting Li[1,2], Ying Gao[1], Chao Peng[3,*], Yong Chen[1,2,4, *]

[1]State Key Laboratory of Molecular Biology, Shanghai Institute of Biochemistry and Cell Biology, Center for Excellence in Molecular Cell Science, Chinese Academy of Sciences, Shanghai, 200031, China.

[2]University of Chinese Academy of Sciences, Beijing, 100049, China.

[3]National Facility for Protein Science in Shanghai, Zhangjiang Lab, Shanghai Advanced Research Institute, Chinese Academy of Science, Shanghai, 201210, China.

[4]School of Life Science and Technology, ShanghaiTech University, Shanghai, 201210, China

[#]These authors contributed equally to this work: Duo Wang, Fuxiang Yan, Ping Wu

*Correspondence: pengchao@sari.ac.cn and yongchen@sibcb.ac.cn

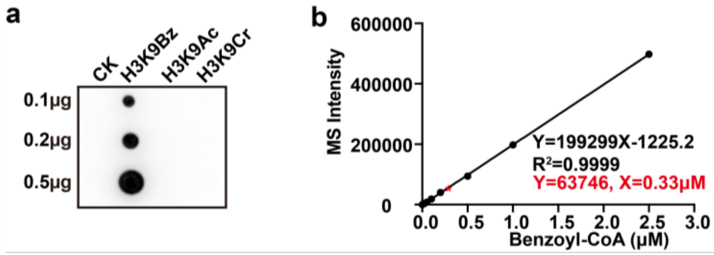


**Supplementary figure 1. Identification of histone Kbz in *S. cerevisiae*.**

a. Dot blot assay using H3K9ac/cr/bz peptides to confirm the specificity of a pan anti-Kbz antibody. Source data are provided in the Source Data file.

b. HPLC-MS/MS analysis of cellular benzoyl-CoA levels extracted from BY4742 cells treated with 10 mM sodium benzoate for 6 h. The standard curve was obtained with various concentrations of benzoyl-CoA standards. The linear fitting between corresponding peak areas and benzoyl-CoA concentrations was achieved ($R^2$=0.9999). The content of benzoyl-CoA is indicated with a red asterisk. The data is from a single experiment. Source data are provided in the Source Data file.

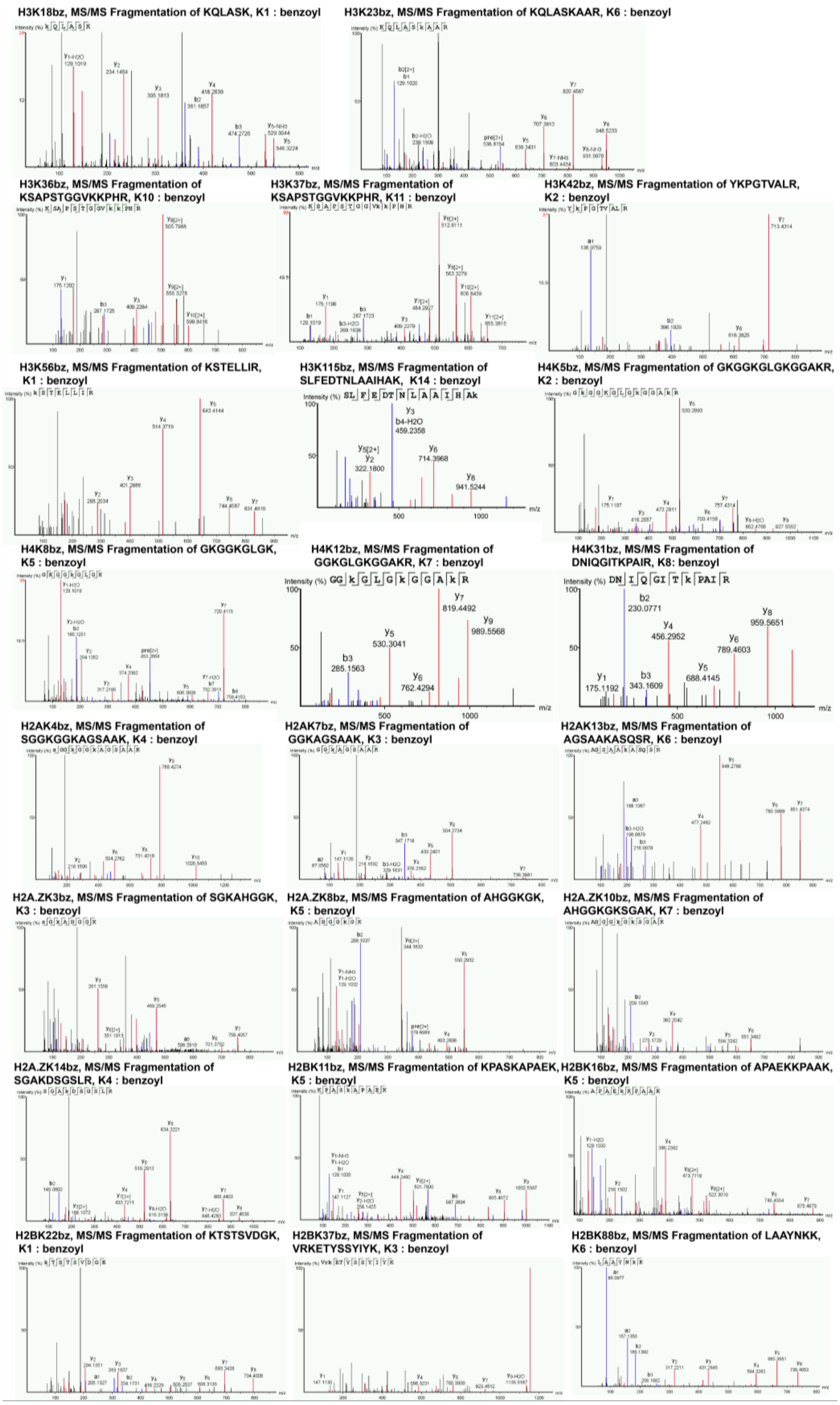


**Supplementary figure 2. The MS/MS spectra of Kbz sites in *S. cerevisiae*.** Predicted b- and y-type ions are listed above and below the peptide sequence, respectively. Matched ions are labeled in the spectra.

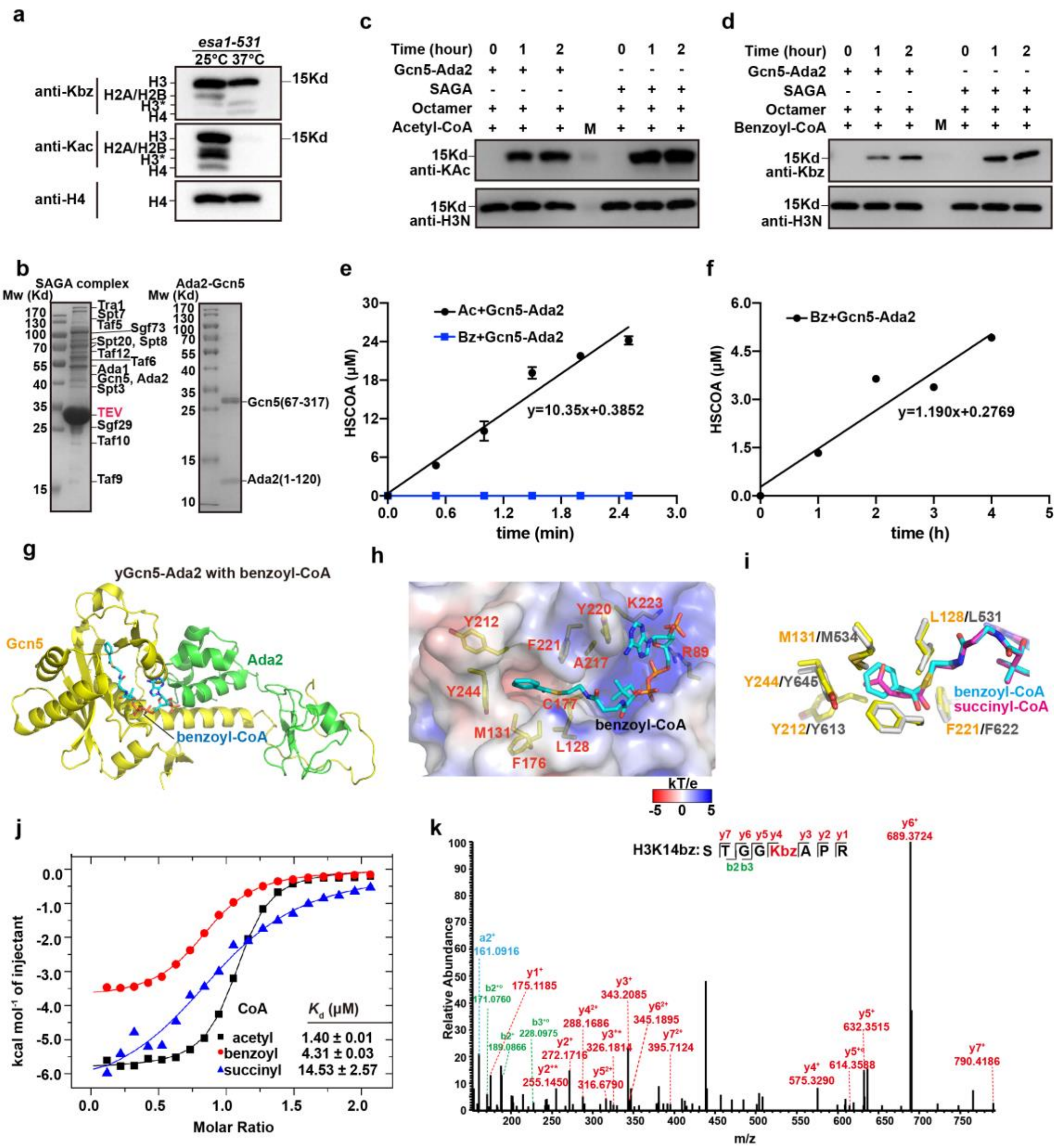


**Supplementary figure 3. Identification of histone benzoyltransferase in *S. cerevisiae*.**

a. The histone acetylation and benzoylation levels of the *esa1* temperature-sensitive mutant *esa1-531* in the presence of 10 mM sodium benzoate. At the permissive temperature (25°C), histones have strong Kac and Kbz levels. At the non-permissive temperature (37°C), acetylation is completely lost, while H3 benzoylation is slightly reduced and H2A/H2B benzoylation is severely decreased. Source data for panels a-f are provided in the Source Data file.

b. SDS-PAGE shows the intact SAGA complex (left) and Ada2-Gcn5 subcomplex (right). TEV in red is a protease used in purification.

c. The Ada2-Gcn5 subcomplex and SAGA complex could catalyze acetylation of the histone octamer in vitro. The reaction products were detected by western blot analysis using a pan anti-Kac antibody.

d. The Ada2-Gcn5 subcomplex and SAGA complex could catalyze benzoylation of the histone octamer in vitro. The reaction products were detected by western blot analysis using a pan anti-Kbz antibody. The catalytic rate of the SAGA complex is higher than the Ada2-Gcn5 subcomplex.

e. The acylation activity assay was performed by measuring the generation of HS-CoA by DNTB. Rates of catalysis by Gcn5-Ada2 (2 μM) were measured using acetyl-CoA or benzoyl-CoA (500 μM) and N-terminal histone H3 peptide (250 μM) containing the sequence ARTKQTARKSTGGKAPRKY. The acetylation rate at the current reaction condition was 10.35 μM/min. The benzoylation signal cannot be detected in the 3-min time frame. Data are presented as mean ± SD, n=3 biological independent measurements.

f. Benzoyltransferase activity of Gcn5-Ada2. Gcn5-Ada2 only showed detectable benzoylation activity after a long-time reaction (more than 1 hour). At the current reaction condition, the benzoylation rate was 1.19 μM/h, which was only 1/520 of the acetylation rate. This result is from a single experiment without repeat.

g. A modeled structure of the benzoyl-CoA-bound Gcn5-Ada2 complex. Benzoyl-CoA, Gcn5, and Ada2 are shown in blue, yellow, and green.

h. The benzoyl-CoA binding pocket of Gcn5. Gcn5 is represented as the surface model colored according to its electrostatic potential (positive potential, blue; negative potential, red). The stick model of benzoyl-CoA is colored in cyan. The Gcn5 residues involved in benzoyl-CoA recognition are shown as stick models in yellow.

i. Comparison of the acyl-binding pockets of yeast Gcn5 (yellow) and human GCN5 (white). The benzoyl-CoA in yeast Gcn5 is colored cyan, and the succinyl-CoA in human GCN5 is colored in magenta.

j. ITC binding curves for the Gcn5-Ada2 complex interaction with benzoyl-CoA (red curve), succinyl-CoA (blue curve), and acetyl-CoA (black curve). The dissociation constants ($K_D$) and their fitting errors are shown.

k. The MS/MS spectra of H3 peptides bearing Kbz modification catalyzed by the Gcn5-Ada2 subcomplex. Predicted b- and y-type ions are listed above and below the peptide sequence, respectively. The circle symbol indicates the neutral loss of water, and the asterisk indicates deamination. Matched ions are labeled in the spectra.

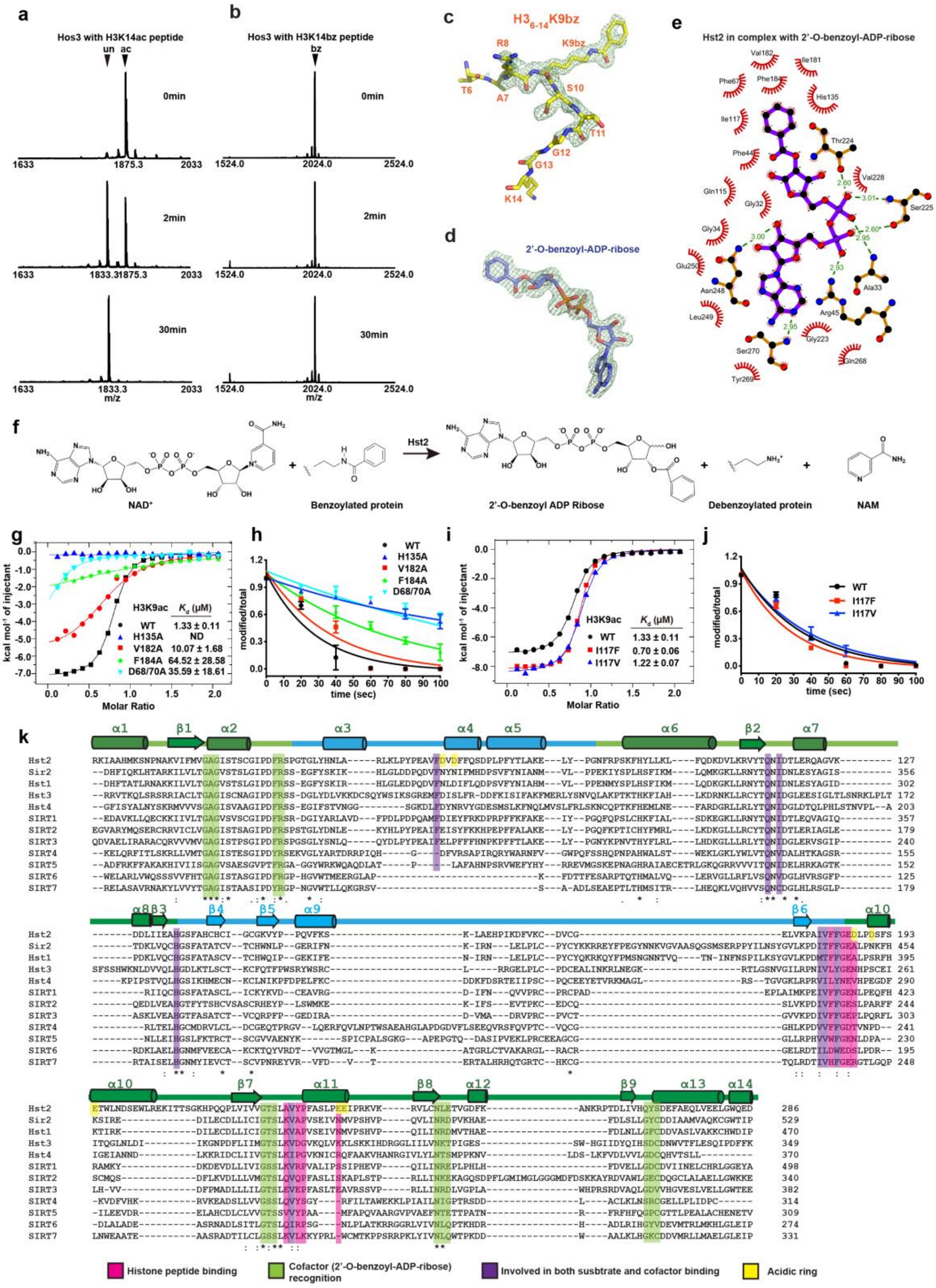


**Supplementary figure 4. Structural basis for Kbz recognition and catalysis of Hst2.**

a. Deacetylase activity of Hos3$_{FL}$ protein (2 μM) with H3K14ac peptide (10 μM, TARKSTGGKacAPRKQLAY) shown by the representative MALDI-TOF spectra at 0, 2, and 30 min. The peaks for H3K14ac (ac, m/z 1,875) and unmodified (un, m/z 1,833) products are labeled.

b. Debenzoylase activity of $Hos3_{FL}$ protein (2 μM) with H3K14bz peptide (10 μM, TARKSTGGKbzAPRKQLASY) shown by the representative MALDI-TOF spectra at 0,2, and 30 min. The peaks for H3K14bz (bz, m/z 2,024) are labeled. Hos3 could not remove the benzoylation group in vitro.

c. The omit map, contoured at the 3 σ level, is shown for the $H3_{6\text{-}14}$ K9bz peptide.

d. The omit map, contoured at the 3 σ level, is shown for the cofactor product of 2’-O-benzoyl-ADP-ribose.

e. The detailed interaction network between Hst2 and 2’-O-benzoyl-ADP-ribose plotted by LigPlot$^{+}$. Eyelash represents hydrophobic contacts. Hydrogen bonding interactions are shown as green dashed lines.

f. The proposed debenzoylation process catalyzed by Hst2.

g. ITC measurements reveal that Hst2 mutations also weaken the Hst2-H3K9ac interaction.

h. MALDI-TOF-based deacetylase assays show that Hst2 mutations also decrease the deacetylase activity of Hst2 for H3K9ac peptides. Data are presented as mean ± SD, n=3 biological independent measurements. Source data f are provided in the Source Data file.

i. ITC results reveal that the I117F and I117V mutations of Hst2 only slightly affected the binding affinities of Hst2 with H3K9ac peptides. The dissociation constant ($K_D$) and their fitting errors are shown.

j. MALDI-TOF-based deacetylase assays show that Hst2 I117F and I117V mutants slightly changed the deacetylation activity. The binding affinity between Hst2 mutants and H3K9ac peptide is correlated to the corresponding deacetylase activity. Data are presented as mean ± SD, n=3 biological independent measurements. Source data are provided in the Source Data file.

k. Sequence alignment of Sirtuin-family HDACs from yeast and *Homo sapiens*. Secondary structure assignments based on the Hst2 structure are shown as cylinders (α-helices) and arrows (β strands) above the sequences. Residues involved in histone peptide binding, 2’-O-benzoyl-ADP-ribose recognition, and both substrate and cofactor binding are highlighted in magenta, green and purple, respectively. The acidic ring is formed by a series of Hst2 negatively-charged residues, which are labeled in yellow.

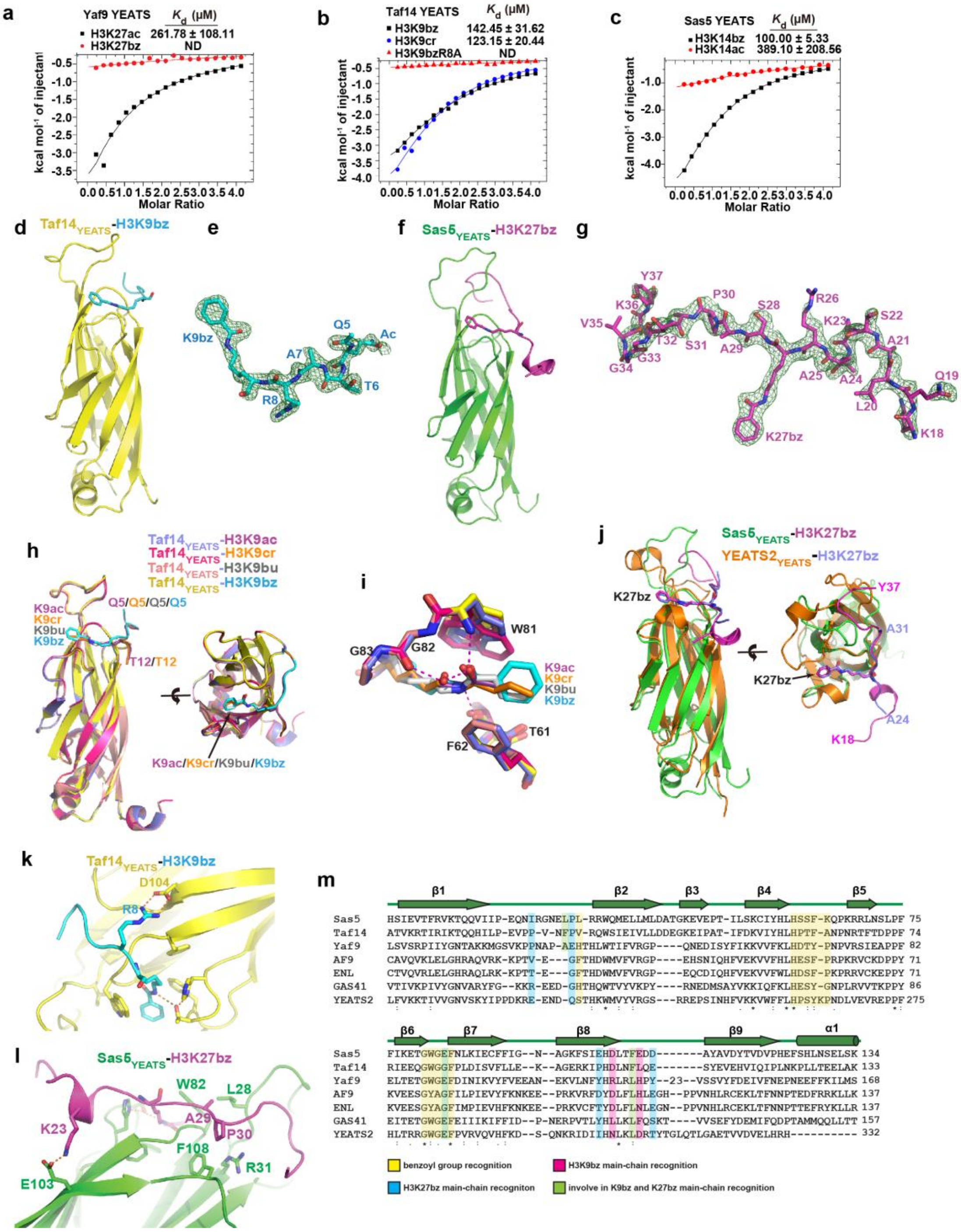


**Supplementary figure 5. Crystal structure of Taf14$_{YEATS}$-H3K9bz and Sas5$_{YEATS}$-H3K27bz complexes.**

a. ITC results reveal that Yaf9$_{YEATS}$ could bind to H3K27ac peptide (Kd = 261 μM) but not H3K27bz peptide.

b. ITC results show that Taf14$_{YEATS}$ has similar binding affinities to H3K9bz and H3K9cr peptides. The H3K9bzR8A mutation significantly decreased the interaction between Taf14 and H3 peptides.

c. ITC results show that $Sas5_{YEATS}$ exhibited the binding preference for Kbz over Kac peptides.

d. The overall structure of the $Taf14_{YEATS}$-H3K9bz complex. The $Taf14_{YEATS}$ is colored in yellow, and the H3K9bz peptide is colored in cyan.

e. The omit map, contoured at the 3 σ level, is shown for the $H3_{5\text{-}9}$K9bz peptide.

f. The overall structure of the $Sas5_{YEATS}$-H3K27bz complex. The $Sas5_{YEATS}$ is colored in green, and the H3K27bz peptide is colored in purple.

g. The omit map, contoured at the 3 σ level, is shown for the $H3_{18\text{-}36}$K27bz peptide. All residues have well-defined electron density, including the extra Y37 for protein quantification.

h. Superimposition of four $Taf14_{YEATS}$-H3 acylated complex structures, including Taf14-H3K9bz, Taf14-H3K9ac (PDB: 5D7E), Taf14-H3K9cr (PDB: 5IOK), and Taf14-H3K9bu (PDB: 6MIQ). The overall configuration of $Taf14_{YEATS}$ with H3K9bz is almost identical to the other three complexes.

i. The detailed interaction network between $Taf14_{YEATS}$ and the K9ac/K9cr/K9bu/K9bz groups. The binding mode of $Taf14_{YEATS}$ with acylated H3 peptides is identical, except for the structural rearrangement of W81.

j. Superimposition of $Sas5_{YEATS}$-H3K27bz and $YEATS2_{YEATS}$-H3K27bz complex structures.

k. The detailed interaction network between $Taf14_{YEATS}$ and H3K9bz peptide. H3 R8 forms salt bridges with Taf14 D104.

l. The detailed interaction network between $Sas5_{YEATS}$ and H3K27bz peptide. H3 A29 and P30 make extensive hydrophobic contacts with a hydrophobic groove composed of Sas5 L28, R31, W82, and F108.

m. Sequence alignment of the YEATS domains from yeast and *Homo sapiens*. Secondary structure assignments based on the Sas5 structure are shown as cylinders (α-helices) and arrows (β strands) above the sequences. Residues involved in benzoyl group recognition, H3K9bz peptide main-chain recognition, H3K27bz peptide main-chain recognition, and both K9bz and K27bz main-chain recognition are highlighted in yellow, magenta, blue, and green, respectively.

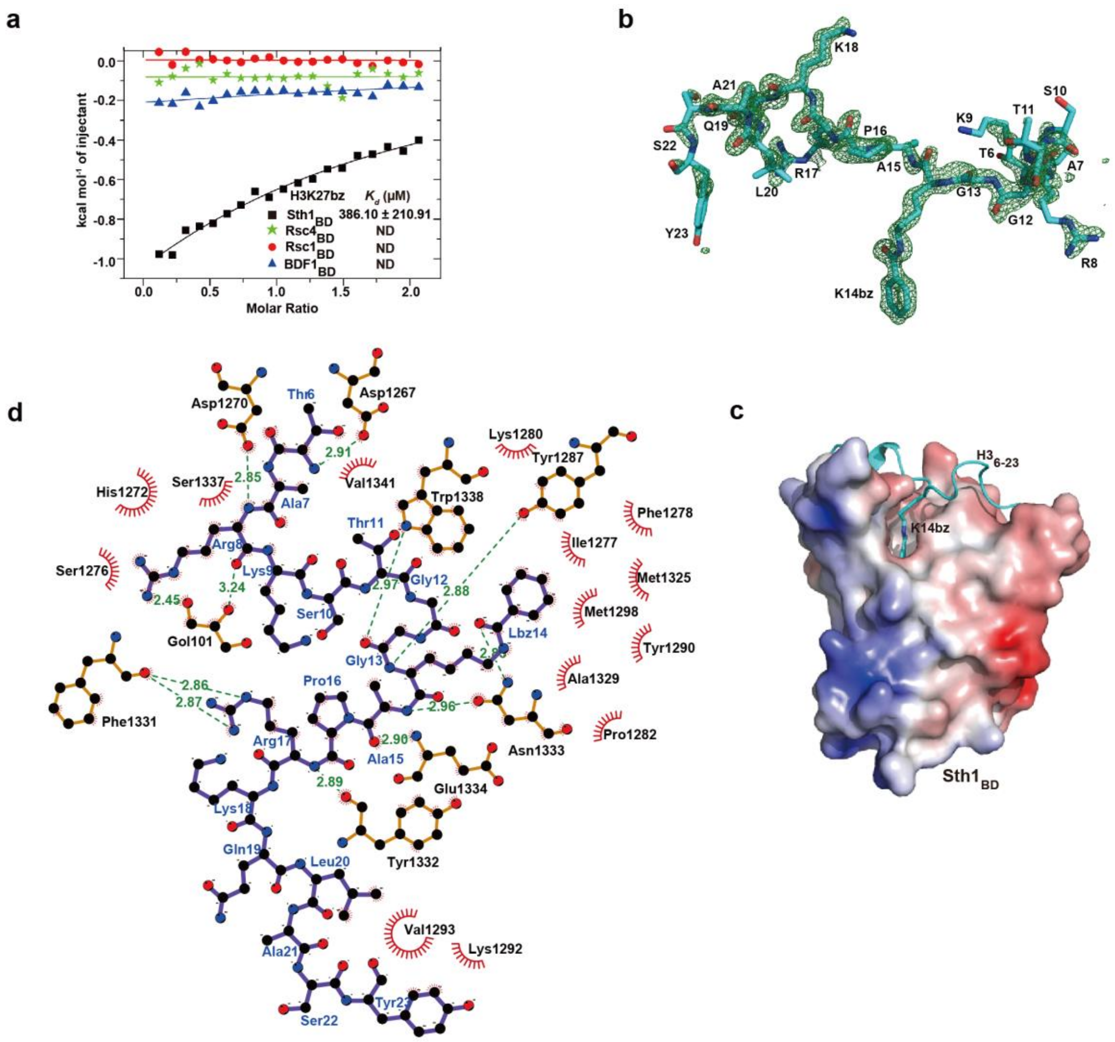


**Supplementary figure 6. Crystal structure of $Sth1_{BD}$-H3K14bz complex.**

a. ITC analyses show that $Sth1_{BD}$ exhibited a robust interaction with the H3K27bz peptide (Kd = 386 μM).

b. The omit map, contoured at the 3 σ level, is shown for the $H3_{6-23}$K14bz peptide. The H3 peptide was modeled including residues 6 to 22, including an extra tyrosine residue (Y23) at the C-terminus designed for concentration determination by UV spectrometry

c. $H3_{6-23}$K14bz sits in a relatively hydrophobic groove of $Sth1_{BD}$ shown in the surface representation. $Sth1_{BD}$ is represented as the surface model colored according to its electrostatic potential (positive potential, blue; negative potential, red).

d. The detailed interaction network between $Sth1_{BD}$ and $H3_{6-23}$K14bz plotted by LigPlot$^{+}$. Eyelash represents hydrophobic contacts. Salt-bridges and hydrogen bonding interactions are shown as green dashed lines.

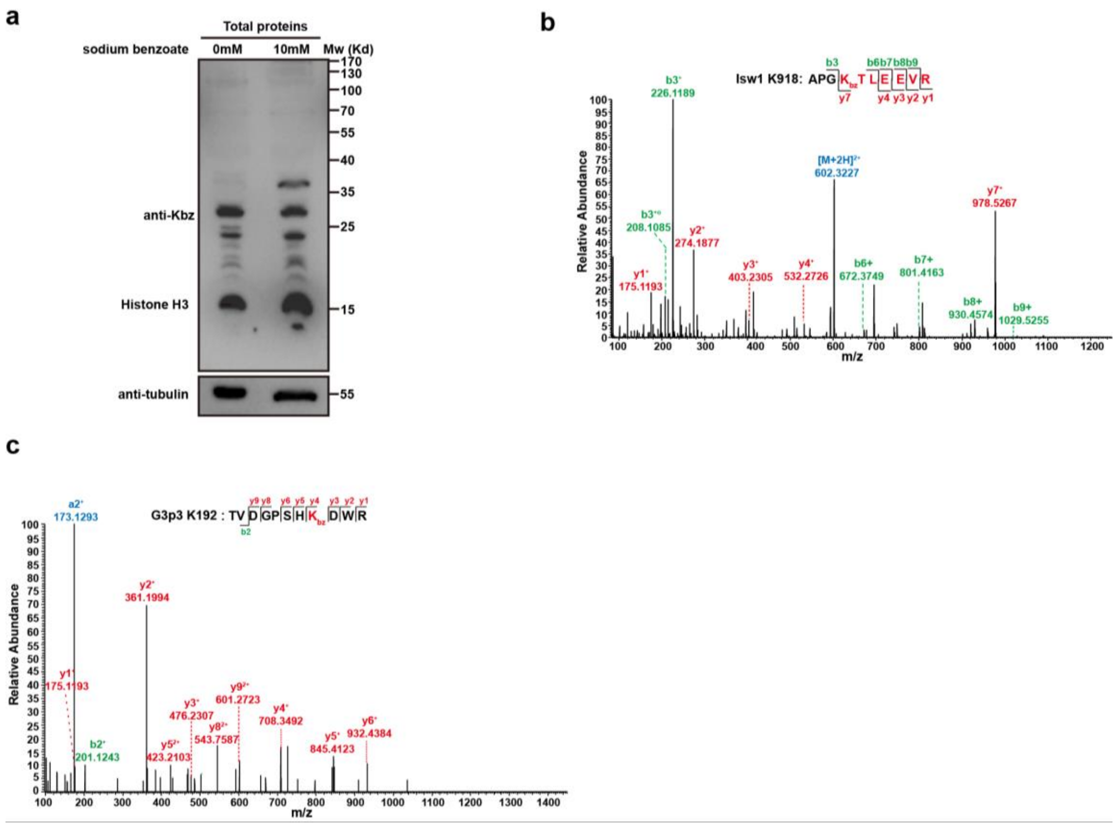


**Supplementary figure7. Representative MS/MS spectra of non-histone $K_{bz}$ sites.**

a. Sodium benzoate treatment significantly enhanced the Kbz level of yeast whole-cell proteins. Source data are provided in the Source Data file.

b-c. Representative MS/MS spectra of identified Kbz peptides. b, Isw1, K918; c, G3p3, K192. Predicted y- and b-type ions are listed above and below the peptide sequence, respectively. The circle symbol indicates the neutral loss of water. Matched ions are labeled in the spectra.

**Supplementary Table1 Primers information**

| Names of constructs | Primer sequence 5’-3’ |
|---|---|
| Hst2$_{1\text{-}357}$ | F:AGAGAACAGATTGGTGGATCCATGTCTGTTTCTACCGCCTCTACA |
| | R:GTGGTGGTGGTGGTGCTCGAGTTATTCTTTAGCGGCTTTTTGTGA |
| Hst2$_{8\text{-}294}$ | F:ACAGAGATGTCGGTTAGGAAGATCG |
| | R:GGATCCACCAATCTGTTCTCTGTGA |
| Taf14$_{2\text{-}137}$ | F:CGGGATCCGTAGCTACAGTAAAAAGAACCATC |
| | R:CCGCTCGAGAGTAGATCCACTTTTCGCAAG |
| Sas5$_{2\text{-}139}$ | F:CGGGATCCGATCATTCAATAGAAGTCACTTTCAG |
| | R:CCGCTCGAGTTACGGGAGATCAAAATATTTAGATAG |
| Sth1$_{1248\text{-}1359}$ | F:GAAGATCTTCGCTTGGGATTTTTCCCACG |
| | R:CCGCTCGAGTTACGAAGAGTGTTCCTTGAACC |
| Ada2$_{1\text{-}120}$ | F:CGGGATCCATGTCAAACAAGTTTCACTGTGACG |
| | R:ACGCGTCGACGGTAATATCAGGTATTGGATAGTATTTGC |
| Gcn5$_{67\text{-}317}$ | F:TTAACTCGAGTCTGGTAAAGAAACCGC |
| | R:AGTCCAGCCGGCTTCTTTCAAGCC |
| SPT20-TAP | F:AAACAGAGCGCAAGTAGCACGCCCTCTTCTACTACAATGTCATCCATGGAAAAGAGAAG |
| | R:TATATATATATATATAAGGAATGATAACTCTATTTAAGTAGATACGACTCACTATAGGG |
| Hst2$_{1\text{-}357}^{D68AD70A}$ | F:GCAGTCGCATTTTTCCAATCGGATCCTTTGCCA |
| | R:GAACACTGCTTCAGGGTACGGC |
| Hst2$_{1\text{-}357}^{H135A}$ | F:GCAGGCAGTTTTGCTCACTGTCAT |
| | R:GGCCTCGATGATCAGATCATCC |
| Hst2$_{1\text{-}357}^{V182A}$ | F:GCATTTTTTGGCGAAGATCTACCGG |
| | R:TATTGCCGGCTTTACCAGTTCA |
| Hst2$_{1\text{-}357}^{F184A}$ | F:GCAGGCGAAGATCTACCGGATTC |

| | |
|---|---|
| | R:AAAAACTATTGCCGGCTTTACCA |
| $Hst2_{1-357}^{I117F}$ | F:TTTGACACTTTAGAAAGACAGGCCGG |
| | R:ATTCTGGGTATATACTCTCTTCAAAACGTCTTTG |
| $Hst2_{1-357}^{I117V}$ | F:GTTGACACTTTAGAAAGACAGGCCGG |
| | R:ATTCTGGGTATATACTCTCTTCAAAACGTCTTTG |

*All primers were synthesized by Biosune company, China.

**Supplementary Table2 Peptides information**

| Names of peptides | Sequence | Source | Purpose |
|---|---|---|---|
| H3K9bz-1 | QTARK(bz)STGGK | Scilight-Peptide | Fig.4a |
| H3K9bz-2 | Ac-QTARK(bz)STGG | Scilight-Peptide | Fig.5d |
| H3K9bz-3 | ARTKQTARK(bz)STGGKAPRKQY | Scilight-Peptide | Fig. 3b, 3d, 3f-g, 4e-f, 4i-j, 5a-c, 5g, Fig. S5b |
| H3K9bzR8A | ARTKQTAAK(bz)STGGKAPRKQY | Scilight-Peptide | Fig.4e, Fig. S5b |
| H3K14bz | TARKSTGGK(bz)APRKQLASY | Scilight-Peptide | Fig. 3f-g, 5a-c, 6a, 6d, Fig. S3b, S5c |
| H3K18bz | STGGKAPRK(bz)QLASKAARY | Scilight-Peptide | Fig. 3f-g, 5a-c, |
| H3K23bz | APRKQLASK(bz)AARKSAPSTY | Scilight-Peptide | Fig. 3f-g, 5a-c |
| H3K27bz | KQLASKAARKbzSAPSTGGVKY | Scilight-Peptide | Fig. 3f-g, 5a-c, 5f, Fig. S5a, S6a |
| H3K9ac-1 | ARTKQTARK(ac)STGGKAPRKQY | Genscript | Fig. 3c |
| H3K9ac-2 | ARTKQTARK(Ac)STGGKAPRKQLAS | Genscript | Fig. 3e, 5j, Fig. S4g-j, |
| H3K9cr | ARTKQTARK(Cr)STGGKAPRKQLATY | Genscript | Fig. S5b |

| | | | |
|---|---|---|---|
| H3K14ac | TARKSTGGK(Ac)APRKQLAY | Genscript | Fig. S4a, S5c |
| H3K27ac | ASKAARK(Ac)SAPSTGGVKKPHRYKPG | Genscript | Fig. 5j, S5a |
| H3P18 | ARTKQTARKSTGGKAPRKY | Genscript | Fig. S3e-f |
