# Supplementary material for: Global profiling of regulatory elements in the histone benzoylation pathway
Source: Nat Commun. 2022 Mar 16;13:1369. doi: 10.1038/s41467-022-29057-2 (PMC8927147; doi:10.1038/s41467-022-29057-2)
Supplement: Supplementary file 3 — Description of Additional Supplementary Files [file 41467_2022_29057_MOESM3_ESM.docx]

**Supplementary Data 1**

**Description:**

**Identification of histone benzoylation sites catalyzed by Gcn5-Ada2 and deposited by non-enzymatic mechanism**

**Supplementary Data 2**

**Description:**

**Global profiling of lysine benzoylation in *S. cerevisiae*.**

**Supplementary Data 3**

**Description:**

**Gene ontology enrichment analysis of benzoylated proteins in *S. cerevisiae*.**
