# Supplementary material for: Global profiling of regulatory elements in the histone benzoylation pathway
Source: Nat Commun. 2022 Mar 16;13:1369. doi: 10.1038/s41467-022-29057-2 (PMC8927147; doi:10.1038/s41467-022-29057-2)
Supplement: Supplementary file 7 — Reporting Summary [file 41467_2022_29057_MOESM7_ESM.pdf]

nature research

Corresponding author(s): Yong Chen

Last updated by author(s): Feb 11, 2022

Reporting Summary

Nature Research wishes to improve the reproducibility of the work that we publish. This form provides structure for consistency and transparency in reporting. For further information on Nature Research policies, see our Editorial Policies and the Editorial Policy Checklist.

Statistics

For all statistical analyses, confirm that the following items are present in the figure legend, table legend, main text, or Methods section.

n/a	Confirmed	
☐	☒	The exact sample size (*n*) for each experimental group/condition, given as a discrete number and unit of measurement
☐	☒	A statement on whether measurements were taken from distinct samples or whether the same sample was measured repeatedly
☐	☒	The statistical test(s) used AND whether they are one- or two-sided<br>*Only common tests should be described solely by name; describe more complex techniques in the Methods section.*
☒	☐	A description of all covariates tested
☒	☐	A description of any assumptions or corrections, such as tests of normality and adjustment for multiple comparisons
☐	☒	A full description of the statistical parameters including central tendency (e.g. means) or other basic estimates (e.g. regression coefficient) AND variation (e.g. standard deviation) or associated estimates of uncertainty (e.g. confidence intervals)
☐	☒	For null hypothesis testing, the test statistic (e.g. *F*, *t*, *r*) with confidence intervals, effect sizes, degrees of freedom and *P* value noted<br>*Give P values as exact values whenever suitable.*
☒	☐	For Bayesian analysis, information on the choice of priors and Markov chain Monte Carlo settings
☒	☐	For hierarchical and complex designs, identification of the appropriate level for tests and full reporting of outcomes
☒	☐	Estimates of effect sizes (e.g. Cohen's *d*, Pearson's *r*), indicating how they were calculated

Our web collection on statistics for biologists contains articles on many of the points above.

Software and code

Policy information about availability of computer code

Data collection	ITC assays were performed using a MicroCal ITC200 system (GE Healthcare); MALDI-TOF assays were carried out using a MALDI-TOF/TOF (SCIEX 5800); The crystal data were collected at the beamline BL19U1/BL18U1 of Shanghai Synchrotron Radiation Facility. The mass samples for Kbz sites identification were loaded onto the EASY-nLC 1200 UHPLC system (ThermoFisher Scientific, San Jose, CA) with a column heater (SCO-2110 pro, China Innovation Instrument Co., Ltd) and analyzed by a Q Exactive mass spectrometer (ThermoFisher Scientific, San Jose, CA); The HPLC-MS/MS analysis of benzoyl-CoA were performed with an Agilent 1290 autosampler (Agilent, USA) coupled to a QTRAP 6500 (Sciex, USA), and the acquired data were extracted and quantified by Skyline v21 (University of Washington, MacCoss Lab, https://skyline.ms) under small molecule mode.; Fluorescent western blots were detected on Odyssey CLx (LI-COR) and quantified by Image Studio (v5.2.5); The spectrometry readings were on BioTek (Synergy NEO).
Data analysis	DAVID 6.8, PyMOL 1.5.0, GraphPad Prism 8.0, phenix 1.18.2, coot 0.8.2, HKL2000 v716, HKL3000 v716, PEAKS Studio 8.5, origin 7.0, Image Studio (Version 5.2.5), Skyline v21 (University of Washington, MacCoss Lab, https://skyline.ms)

For manuscripts utilizing custom algorithms or software that are central to the research but not yet described in published literature, software must be made available to editors and reviewers. We strongly encourage code deposition in a community repository (e.g. GitHub). See the Nature Research guidelines for submitting code & software for further information.

Data

Policy information about availability of data

All manuscripts must include a data availability statement. This statement should provide the following information, where applicable:

- Accession codes, unique identifiers, or web links for publicly available datasets
- A list of figures that have associated raw data
- A description of any restrictions on data availability

The crystal structures have been deposited in the Protein Data Bank with accession code 7F3S (Sth1BD-H3K14bz), 7F4A (Taf14YEATS–H3K9bz), 7F5M (Sas5YEATS–H3K27bz), 7F4E (Hst2-H3K9bz complex), and 7F51 (Hst2-2'-O-Benzoyl-ADP-ribose complex). The mass spectrometry data have been deposited to the ProteomeXchange Consortium via the PRIDE partner repository with the dataset identifier PXD030110 (LC-MS/MS identification of histones Kbz sites catalyzed by non-enzymatic mechanism and by Gcn5-Ada2), PXD030070 (LC-MS/MS identification of histone Kbz sites in yeast), and PXD029997 (global profiling of lysine benzoylation in S. cerevisiae). Subcellular distribution analysis was performed by Uniport (https://www.uniprot.org/). Gene Ontology analysis was performed by DAVID platform 6.8 (https://david.ncifcrf.gov/). Source data are provided with this paper. All other data are available from the corresponding author on request.

Field-specific reporting

Please select the one below that is the best fit for your research. If you are not sure, read the appropriate sections before making your selection.

- [x] Life sciences
- [ ] Behavioural & social sciences
- [ ] Ecological, evolutionary & environmental sciences

For a reference copy of the document with all sections, see nature.com/documents/nr-reporting-summary-flat.pdf

Life sciences study design

All studies must disclose on these points even when the disclosure is negative.

Sample size	No statistical methods were used to predetermine sample size. Sample sizes were decided based on the literature and prior experience in our laboratory. All MALDI-ToF based deacetylation and debenzoylation experiments and the acetylation rate assays of Gcn5-Ada2 subcomplex were performed independent three times. Afterwards, the results of the three experiments were statistically analyzed by GraphPad Prism 8.0 software.
Data exclusions	No data were excluded in this study.
Replication	All ITC experiments were repeated twice, and one representative plot was shown. The WB assays and purification of SAGA and Gcn5-Ada2 subcomplex were performed at least twice. The identification of histone and non-histone Kbz sites were performed at least twice. All MALDI-TOF based deacetylation and debenzoylation experiments and the acetylation rate assays of Gcn5-Ada2 subcomplex were repeat independent three times. The MS/MS identification of histone Kbz sites catalyzed by non-enzymatic or Gcn5-Ada2 subcomplex assays shown in Fig. 2d-e, and the benzoylation rate assays shown in Supplementary Fig. 3f were not repeated due to the material limitation.
Randomization	Samples were randomly allocated to different groups.
Blinding	Yeast strains were blinded to group allocation.

Reporting for specific materials, systems and methods

We require information from authors about some types of materials, experimental systems and methods used in many studies. Here, indicate whether each material, system or method listed is relevant to your study. If you are not sure if a list item applies to your research, read the appropriate section before selecting a response.

Materials & experimental systems

n/a	Involved in the study	
[ ]	[x]	Antibodies
[ ]	[x]	Eukaryotic cell lines
[x]	[ ]	Palaeontology and archaeology
[x]	[ ]	Animals and other organisms
[x]	[ ]	Human research participants
[x]	[ ]	Clinical data
[x]	[ ]	Dual use research of concern

Methods

n/a	Involved in the study	
[x]	[ ]	ChIP-seq
[x]	[ ]	Flow cytometry
[x]	[ ]	MRI-based neuroimaging

Antibodies

Antibodies used	The HRP secondary antibody were used: Peroxidase AffiniPure Goat Anti-Mouse IgG (Jackson ImmunoResearch, 1:5000, 115-035-003), Peroxidase AffiniPure Goat Anti-Rabbit IgG (Jackson ImmunoResearch, 1:5000, 111-035-003). And anti-Kbz (Anti-

	Benzoyllysine Mouse mAb, PTM Biolabs Inc., PTM-762, 1:1000, Lot# 1314381619I926), anti-Kac (Cell signal, 9441S, 1:1000, Lot# 14), anti-H3N (Active motif, anti-Histone 3 Mouse mAb, 39763, 1:700, Lot# 20418023), anti-H4 (Active motif, anti-Histone 4 Mouse mAb, 61521, 1:3000, Lot# 31416005), and anti-tubulin (Sigma, anti-tubulin Mouse mAb, T6199, 1:3000, Lot# 048M4751V) antibodies were used in this study. The following fluorescent secondary antibodies were used: IRDye 800CW goat anti-rabbit IgG (LI-COR P/N 923-32211, 1:5000, Lot# D00304-15) and IRDye 680RD goat anti-Mouse IgG (LI-COR P/N 926-68070, 1:10000, Lot# D01014-04).
Validation	PTM-762 is monoclonal antibody detecting Kbz. Host: Mouse. PTM-762 has been successfully used in Western Blot, and does not possess Immunogen species.<br>Cell signal 9441S is a polycolonal antibody and detects proteins posttranslationally modified by acetylation on the epsilon-amine groups of lysine residues. Host: Rabbit. Polyclonal antibodies are produced by immunizing animals with a synthetic acetylated lysine-containing peptide. Species Reactivity: All Species Expected; 9441S has been successfully used in Western Blot, Elisa, CHip, IP, IF.<br>Active Motif 39763 is monoclonal antibody detecting Histone 3. Host: Mouse. This Histone H3 antibody (mAb) was raised against a peptide containing the N-terminus of histone H3. Reactivity:Human, Wide Range Predicted; 39763 has been successfully used in Western Blot, CHIP, CHIP-seq, ICC, IF.<br>Active Motif 61521 is monoclonal antibody detecting Histone 4. Host:Mouse. This antibody was raised against a synthetic peptide containing human Histone H4. Reactivity:Human, Wide Range Predicted. 61521 has been successfully used in Western Blot, CHIP, IHC.<br>Sigma T6199 is monoclonal primary antibody detecting tubulin. Host:Mouse. This antibody is DM1A produced by fusion of mouse myeloma cells (NS1) and purified chicken brain tubulin immune BALB/c mouse spleen cells Hybridomas are derived. Reactivity: bovine, rat, yeast, human, mouse, chicken, fungi, amphibian. T6199 has been successfully used in Western Blot,IF.

Eukaryotic cell lines

Policy information about cell lines

Cell line source(s)	BY4742, HAT ko and HDAC ko yeast strains from Jinqiu Zhou lab; esa1-531 strain from Junbiao Dai Lab.
Authentication	*Describe the authentication procedures for each cell line used OR declare that none of the cell lines used were authenticated.*
Mycoplasma contamination	*Confirm that all cell lines tested negative for mycoplasma contamination OR describe the results of the testing for mycoplasma contamination OR declare that the cell lines were not tested for mycoplasma contamination.*
Commonly misidentified lines (See ICLAC register)	*Name any commonly misidentified cell lines used in the study and provide a rationale for their use.*
